# Endoscopic trans-eustachian tube approach: identifying the precise landmarks, a novel radiological and anatomical evaluation

**DOI:** 10.1007/s00276-024-03344-7

**Published:** 2024-03-26

**Authors:** Ali Karadag, Mustafa Eren Yuncu, Erik H. Middlebrooks, Necmettin Tanriover

**Affiliations:** 1 Izmir Faculty of Medicine, Department of Neurosurgery, University of Health Sciences, Izmir, Turkey; 2 Department of Neurosurgery, Izmir City Hospital, Laka, Bornova / Izmir, 35040 Turkey; 3 Department of Neurosurgery, Mayo Clinic, Jacksonville, FL USA; 4 Department of Radiology, Mayo Clinic, Jacksonville, FL USA; 5 Cerrahpasa Faculty of Medicine, Department of Neurosurgery, Istanbul University – Cerrahpasa, Istanbul, Turkey; 6 Cerrahpasa Faculty of Medicine, Department of Neurosurgery, Microsurgical Neuroanatomy Laboratory, Istanbul University – Cerrahpasa, Istanbul, Turkey

**Keywords:** Endoscopic, Anatomy, Eustachian tube, Skull base, Infratemporal fossa

## Abstract

**Purpose:**

The endoscopic trans-eustachian approach (ETETA) is a less invasive approach to the infratemporal fossa (ITF), providing superior exposure compared to traditional transcranial approaches. The anatomy of the pharyngotympanic (eustachian) tube and adjacent neurovascular structures is complex and requires in-depth knowledge to safely perform this approach. We present a cadaveric and radiological assessment of critical anatomic considerations for ETETA.

**Methods:**

Six adult cadaveric heads were dissected alongside examination of 50 paranasal sinus CT scans. Key anatomic relationships of the pharyngotympanic tube and adjacent structures were qualitatively and quantitatively evaluated. Descriptive statistics were performed for quantitative data.

**Results:**

Anatomical and radiological measurements showed lateralization of the pharyngotympanic tube allows access to the ITF. The pharyngotympanic tube has bony and cartilaginous parts with the junction formed by the sphenoid spine and foramen spinosum. The bony part and tendon of the tensor tympani muscle were located at the posterior genu of the internal carotid artery. The anterior and inferior wall of the carotid canal was located between the horizontal segment of the internal carotid artery and petrous segment of the cartilaginous pharyngotympanic tube.

**Conclusion:**

The combination of preoperative radiographic assessment and anatomical correlation demonstrates a safe and effective approach to ETETA, which allowed satisfactory visualization of ITF. The morphological evaluation showed that the lateralization of the pharyngotympanic tube and related structures allowed a surgical corridor to reach the ITF. Endoscopic surgery through the pharyngotympanic tube is challenging, and in-depth understanding of the key anatomic relationships is critical for performing this approach.

## Introduction

The complex anatomy of the anterolateral and the middle skull base presents many challenges for surgical access and has led to the development of minimally invasive and endoscopic approaches [11, 14, 24]. Continued advancements in endoscopic transnasal approaches (ETA) to the skull base have expanded applications over the last decades [11, 24]. The ETAs to the infratemporal fossa (ITF) and adjacent skull base are less invasive than the traditional transcranial approaches and an innovative surgical technique to reduce complications and morbidity [4, 24]. Endoscopic approaches provide a suitable surgical corridor with adequate visualization of the region between the anterior wall of the middle ear cavity, petrous apex (PA), and nasopharynx in the ITF if the associated structures and landmarks are navigated effectively [4, 24].

Several approaches to the ITF and PA via the pharyngotympanic tube (PT), also known as the eustachian tube, have been described [8, 21, 22]. Knowledge of the anatomy of the PT is important to perform these approaches [8, 15]. The PT has complex anatomy encompassing bone, mucosa, cartilage, muscle, and neurovascular structures [2, 14, 16]. The PT consists of a medial osseous and a lateral cartilaginous part that can be divided into nasopharyngeal, pterygoid, lacerum, petrous, and osseous segments [22]. The aim of this study was to demonstrate the relevant anatomy of the endoscopic trans-eustachian tube approach (ETETA) using a combination of cadaveric dissection and CT images.

## Methods

### Anatomical dissection

Six formalin-fixed and silicone-injected adult cadaver heads (12 sides) without skull base pathology were dissected. The PT, pterygopalatine plaque, and ITF were stepwise dissected using 0˚ and 30˚ endoscopes. A vernier caliper (0.1 mm precision) (Vernier Software and Technology, Beaverton, OR, USA), a goniometer (for measuring length and angle (precision up to 1 degree)), and Storz endoscope (Karl Storz SE, Tuttlingen, Germany) were used. All the cadaver heads were positioned with 10 degrees of flexion, and the dissections were performed using the position that simulated the orientation in surgery. The surgical landmarks through the ETETA to the ITF were assessed.

Relevant anatomic parameters were measured on the left and right sides. We examined the anterior (ACF) and middle cranial fossa (MCF) anatomy in the context of an ETA, as well as compared the surgical views provided by the ETETA to the ITF. We then assessed quantitative and qualitative features of the PT and associated structures in ETA to the ITF. A Vernier caliper (accurate to 0.1 mm), a goniometer, and manual measuring instrument were used to measure the following parameters.

### Radiologic assessment

**Fig. 1:**
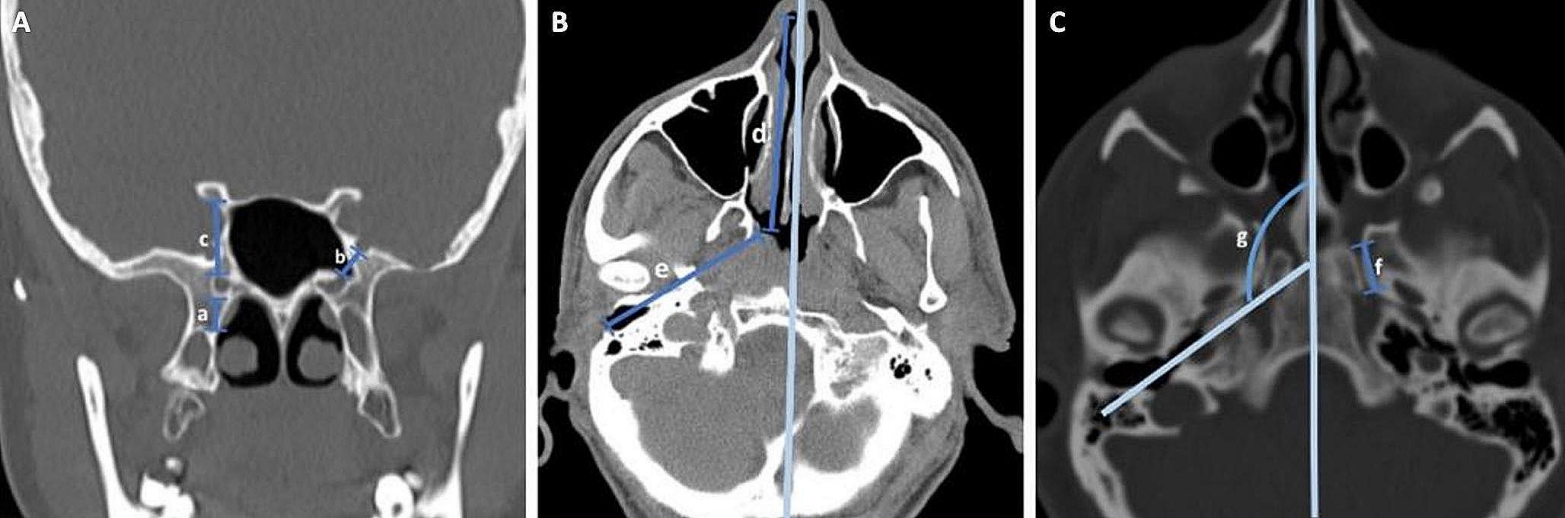
Illustration of radiologic measurements obtained from CT scans. **(A)** Coronal CT showing the distance from vidian canal (VC) to the base of the medial pterygoid plate (a), the distance from the ventral opening of the VC to the foramen rotundum (FR) (coronal plane) (b), and the distance from the ventral VC opening to the optic canal (c). **(B)** Axial CT showing the distance from the nasal vestibule to the PT orifice (d) and the length of VC from anterior to posterior (e). **(C)** Axial CT showing the length of the PT (from the nearest point to the pharynx of the pharyngeal PT orifice to the tympanic orifice) (f) and an obtuse angle between the midline and the oblique line that is parallel to the PT trajectory and passes from pharyngeal PT orifice and tympanic orifice (g)

We retrospectively evaluated a suitable paranasal CT scan from 50 consecutive adult patients without previous history of ACF and MCF fractures or any reported pathology or surgery involving the area of interest. We used the measurement tools of our radiology imaging system (Probel Software, İzmir, Turkey) to measure the relevant parameters, as shown in Fig. 1.

### Statistical analysis

Where appropriate, anatomical and radiological data were expressed as mean, standard deviation ± SD, and range using Prism (GraphPad Software, Inc.) software version 6.0.

## Results

**Fig. 2:**
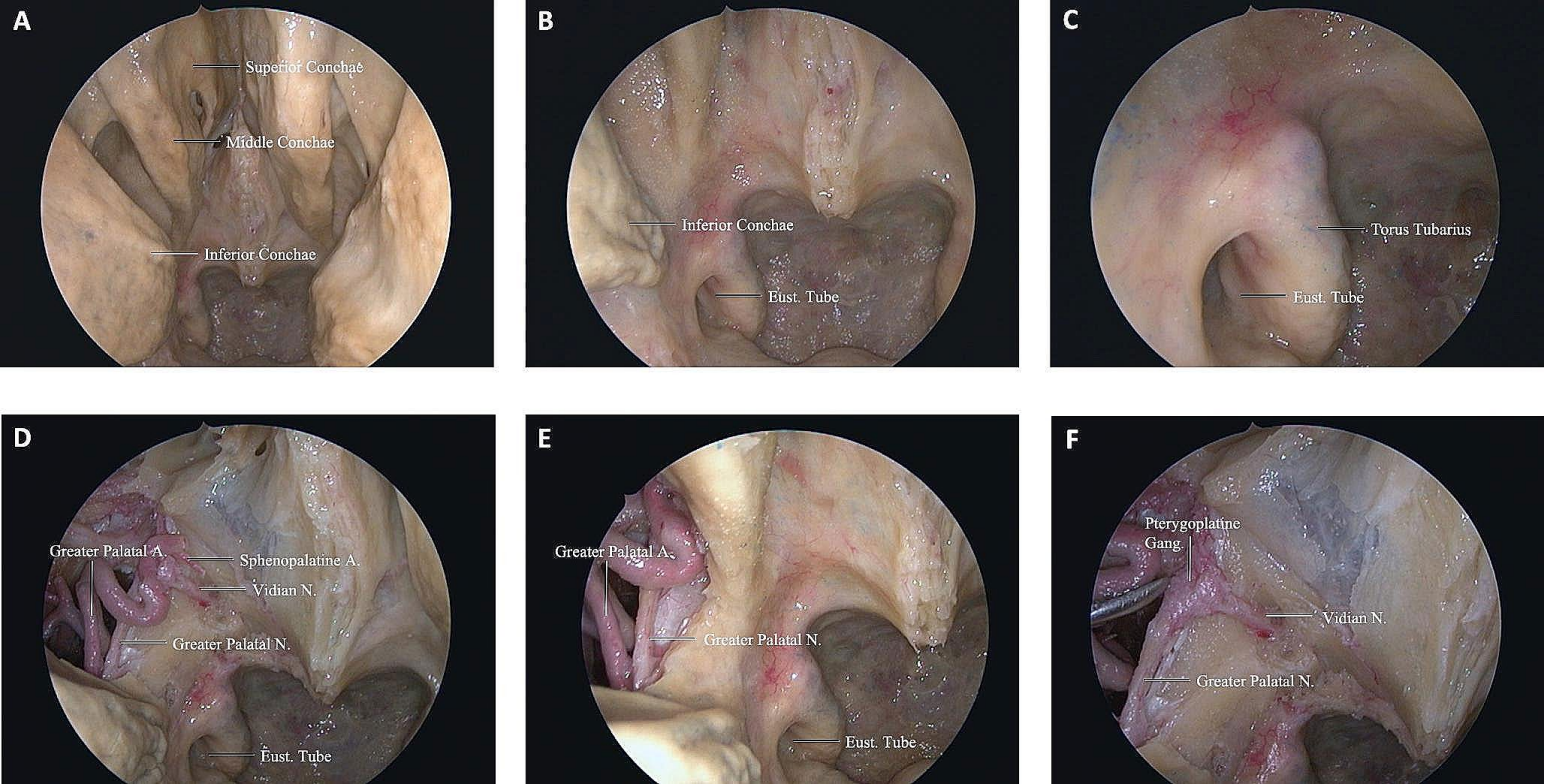
Endoscopic images of ETETA. **(A)** After advancing into the nasal cavity, the lower, middle, and upper turbinates were visualized. **(B)** PT between the middle and inferior turbinates was demonstrated. **(C)** Close-up of Fig. 2B. **(D)** In the endonasal procedure, the sphenopalatine artery should be exposed first. After the sphenopalatine artery was isolated, the lateral wall of the nasal cavity was resected. **(E)** The greater palatal artery and nerve were shown in the lateral wall of the nasal cavity. **(F)** The vidian nerve is shown within the vidian canal

**Fig. 3:**
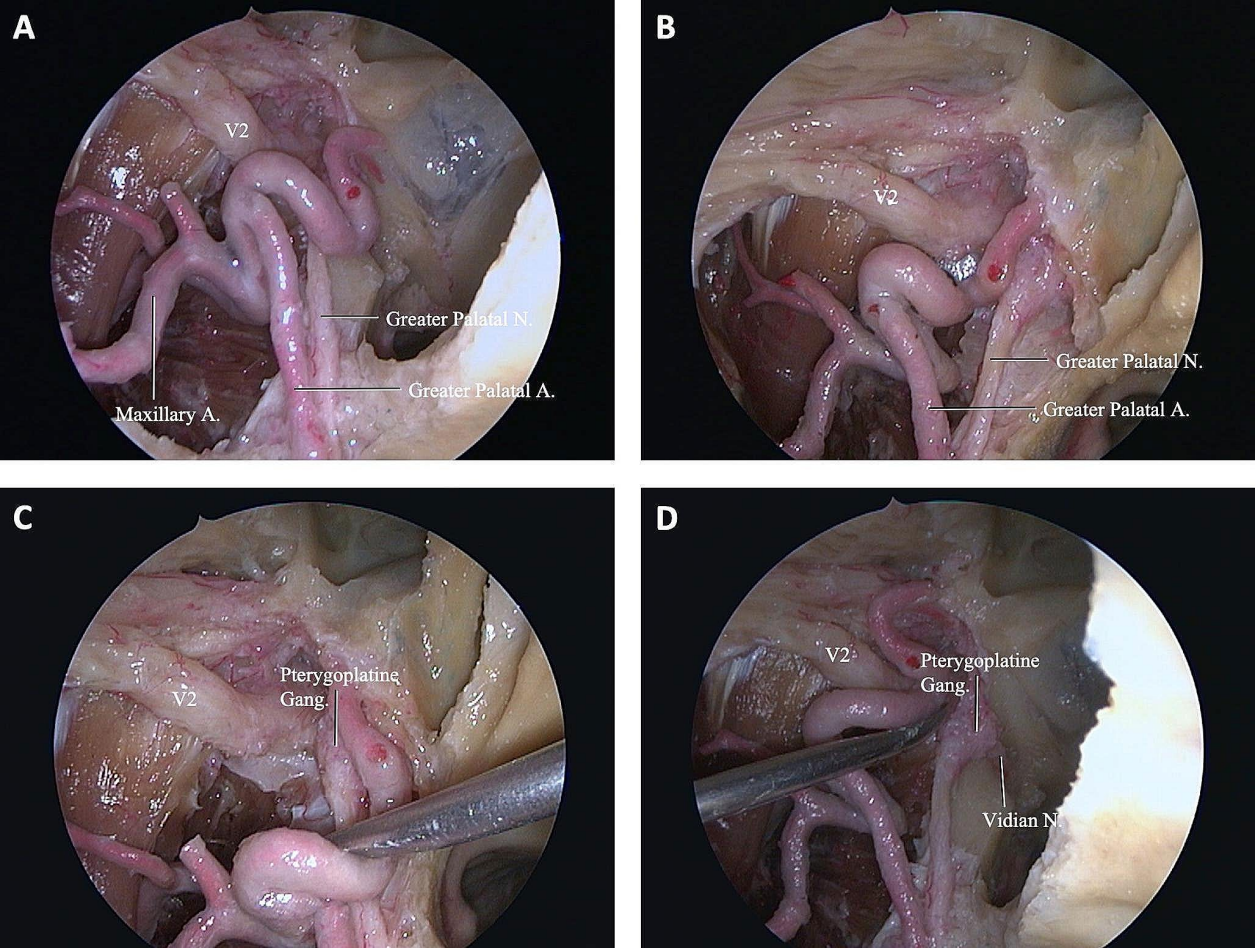
Endoscopic images of ETETA. **(A)** The course of the greater palatal artery and nerve and the sphenopalatine artery is shown. **(B)** Oblique view to Fig. 3A. **(C)** The sphenopalatine artery was retracted medially, showing the pterygopalatine ganglion. **(D)** The sphenopalatine artery was retracted laterally and the pterygopalatine ganglion and vidian nerve were revealed

**Fig. 4:**
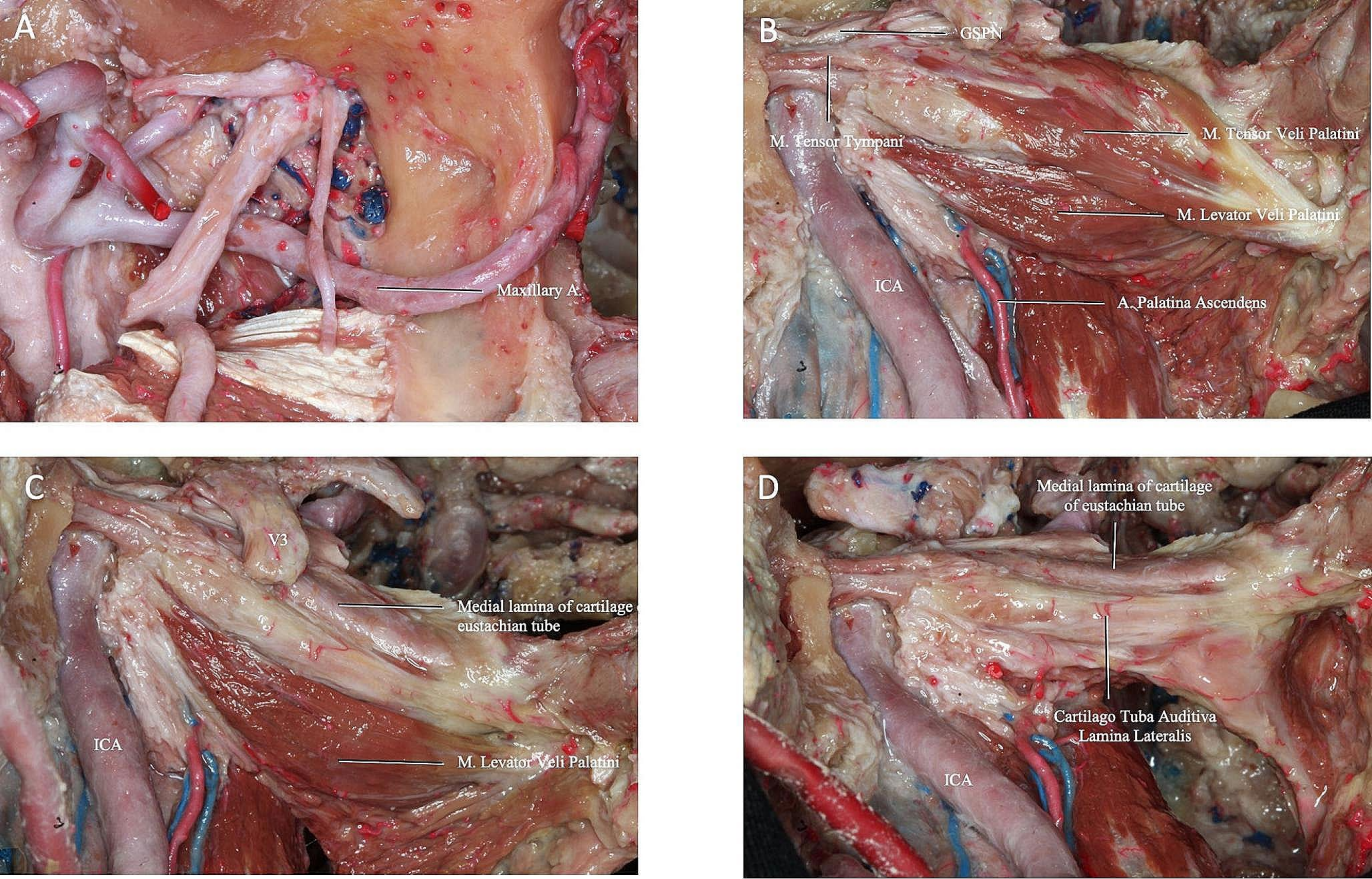
Endoscopic images of ETETA. **(A)** After periosteum removal of the diffuse adipose tissue around the posteromedial maxillary sinus wall and pterygopalatine fossa, the distal segment and branches of the maxillary artery (ascending palatine artery, sphenopalatine artery) were shown. **(B)** The region of PPF with MPMs posteriorly, palatine bone anteromedially, and maxilla anterolaterally is shown. **(C)** FO and V3 can be revealed by resection of the LPM. Resection of V3 may be necessary to expose the entire posterolateral portion of the PT. With ETETA, dissection of TVPM from PT allows observation of the course of PT without compromising V3. **(D)** After the TVPM and LVPM are resected, the cartilaginous laminae and PT are clearly exposed

In all specimens, we were able to endoscopically expose the ACF and MCF from the nasopharynx to the PA, internal carotid artery (ICA), and the ITF space. After progressing into the nasal cavity, the inferior, middle, and superior conchae were visualized, and we focused on the area sits between the middle and inferior conchae (Fig. 2A-C). First, the sphenopalatine artery should be exposed. After isolating the sphenopalatine artery (lateralize or cauterize), the next step was removal of the lateral wall of the nasal cavity down to the level of the nasal floor (Fig. 2D-F). The mean width (superior-inferior) of the surgical corridor between the inferior and middle concha without concha resection was 10.1 ± 0.92 mm on the left and 10.56 ± 0.55 mm on the right, which shows the entry point of the ETETA and width of the surgical window. Also, the mean shortest distance from the nasal vestibule to the PT orifice was 76 ± 4.58 mm on the left and 75 ± 3.6 mm in the right nostril. The mean length of the PT was 41.7 ± 2.3 mm on the left and 42.7 ± 0.32 mm on the right side (Table 1). The sphenopalatine artery was located between the orbita and ethmoidal crest, anterior to the vidian nerve (VN) and pterygopalatine ganglion (Fig. 3). The mean direct distance from the ventral opening of the vidian canal (VC) to the left PT orifice was 22.9 ± 0.51 mm and 22.7 ± 0.65 mm on the left and right side, respectively (Table 1). After removing the periosteum of the posteromedial maxillary sinus wall, the distal segment of the internal maxillary artery (IMA) and its branches (ascending palatine artery, sphenopalatine artery) were identified (Fig. 4A). At the level of VN, with the vascular compartment of the pterygopalatine fossa (PPF) lateralized, the pterygopalatine ganglion, greater palatine nerve, lesser palatine nerve, infraorbital nerve, and maxillary nerve (V2) at the foramen rotundum (FR) were easily visualized and manipulated. It is critical to appreciate the distance of dissection to the FR to avoid neurovascular injury during drilling. We found the PPF was limited by the medial pterygoid muscles (MPM) posteriorly, the palatine bone anteromedially, and the maxilla anterolaterally (Fig. 4B). The VC was directed posteriorly toward the second genu of the ICA. Once the VC was exposed, the base of the pterygopalatine plates (PTPs) was identified immediately laterally. The VN was transected just proximal to its junction with the sphenopalatine ganglion to expose the lateral pterygoid plate (LPP) (Fig. 4A, B). The mean shortest distance from the ventral VC opening to the optic canal was 22.4 ± 0.9 mm on the left and 21.4 ± 0.55 mm on the right side (Table 1). Partial drilling of the pterygoid process around the VC in anterior-posterior direction created the window needed for accessing the deep target regions (ITF and PA). The IMA, V3, TVPM, LVPM, and PT can be exposed in front of the ITF. Foramen ovale (FO) and the mandibular nerve (V3) can be exposed with initial dissection of the lateral pterygoid muscle (LPM). V3 was an important landmark to locate the post-styloid compartment, as it was always just anterior to this space. The cartilaginous part of PT ran anteromedial to the FO. Dissection of the TVPM from the PT allowed the course of the PT to be observed without sacrificing the V3 (Fig. 4B, C).

**Table 1:** Morphometric measurements of Eustachian tube and its associated anatomical structures in the infratemporal fossa

N: 6	Average ± SD (min, max) left side	Average ± SD (min, max) right side
The length of the PT (from the nearest point to the pharynx of the pharyngeal PT orifice to the tympanic orifice) (mm)	41.7 ± 2.3 (39.5, 44.1)	42.7 ± 0.32 (42.5, 43.1)
The shortest distance from the nasal vestibule to the PT orifice (mm)	76 ± 4.58 (71, 80)	75 ± 3.6 (72, 79)
The width (superior-inferior) of the surgical corridor between the inferior and middle concha without concha resection lateral wall of the nasal cavity (mm)	10.1 ± 0.92 (9.1, 10.9)	10.56 ± 0.55 (10.2, 11.2)
The shortest distance from the ventral VC opening to the optic canal (mm)	22.4 ± 0.9 (21.5, 23.3)	21.4 ± 0.55 (21.1, 22.1)
The direct distance from the ventral opening of the VC to the left PT orifice (mm)	22.9 ± 0.51 (22.4, 23.1)	22.7 ± 0.65 (22.1, 23.4)
An obtuse angle between the midline and the oblique line which is parallel to the PT trajectory and passes from pharyngeal PT orifice and tympanic orifice (axial plane) (degree)	119.6 ± 3.81 (116.6, 123.9)	119.8 ± 4.45 (115.5, 124.4)

**Fig. 5:**
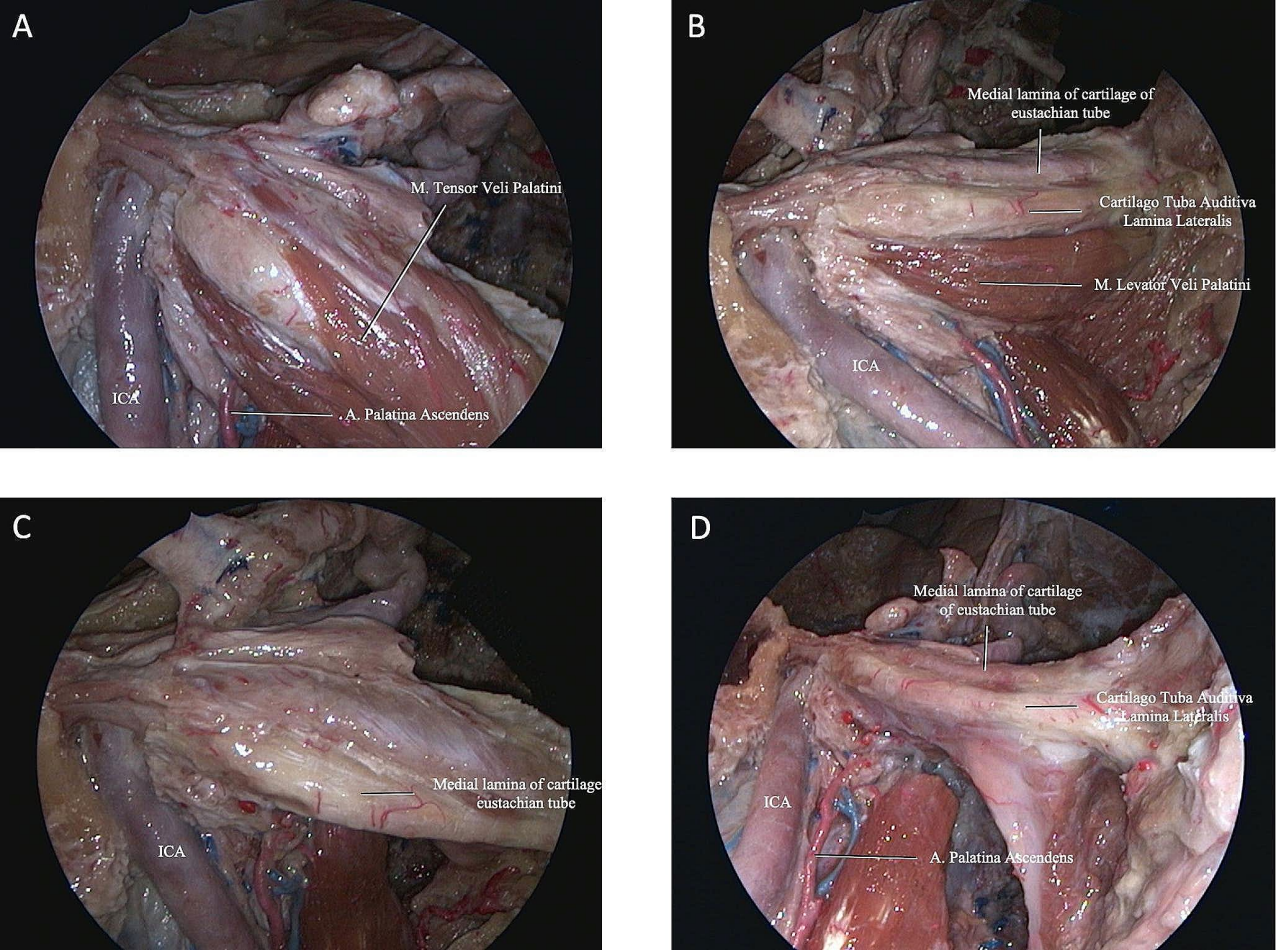
**(A)** Dissection stages were continued with endoscope guidance. PT was demonstrated anteromedial to PphICA. **(B)** Resection of the TVPM revealed the cartilaginous auditory tube lamina medialis and lateralis. **(C)** Resection of the LVPM yielded the anterolateral skull base attachment of the PT. **(D)** Oblique view to Fig. 5C.

The cartilaginous PT has a non-cartilaginous gap located inferolaterally between the medial and lateral cartilaginous laminae, which is covered by the tensor veli palatini muscle (TVPM), levator veli palatini muscle (LVPM), and the lateral fat pad. After removing the muscles and fat pad, the cartilaginous laminas and PT were exposed (Fig. 4D). The dissection was continued using the endoscope. The ITF was bounded superiorly by the floor of the MCF and anteriorly by the maxilla [5]. The pre-styloid compartment of ITF has a fat-containing space and is located between the MPM and TVPM. In addition, the ICA, internal jugular vein (IJV), and lower cranial nerves (IX through XII) are located in the post-styloid compartment (Fig. 5) [5].

Next, PT was observed anterior and medial to the parapharyngeal ICA (PphICA). Laterally translocating or resecting these neurovascular structures showed, in order, the MPM, TVPM, LVPM, and PT (Fig. 5A, B). MPM and the TVPM were closely related; therefore, complete separation is not always possible. Reaching the anterolateral skull base attachment of the PT was possible with resection of the TVPM from the anterolateral surface of the PT (Fig. 5A). PT was fully preserved to the PphICA, which was located between the styloid process and Rosenmüller’s fossa. The cartilaginous part of PT and torus tubarius (TT) inserted in the posterior border of the medial pterygoid process anteriorly (Fig. 5C, D) and the clivus and foramen lacerum (FL) posteriorly. From the TT, the cartilaginous PT extended posterosuperiorly until reaching its bony canal at the cranial base [14–16]. The TVPM and LVPM inserted in the anterior and inferior aspect of the cartilaginous PT. We found the PphICA was located at the junction of the bony and cartilaginous part of the PT, which corresponds to the most superior aspect of the insertion of the LVPM. The tensor tympani muscle (TTM) was located within a bony semicanal located superior and parallel to the semicanal of the PT (Fig. 5A and Fig. 4B). After drilling this area, the TTM anterior to the cartilaginous part of the PT was exposed. The TTM turns across the tympanic cavity and inserts into the medial margin of the handle of the malleus. The TVPM was located medial to the MPM and had both a bony insertion anterior to the carotid foramen and a cartilaginous insertion on the medial lamina of the cartilaginous PT. It originated from the scaphoid fossa, sphenoid spine, and lateral lamina of the cartilaginous PT, ran vertically down between the MPM and MPP and ended with the tendon winding around the pterygoid hamulus approximately parallel to the lumen of the PT.

**Fig. 6:**
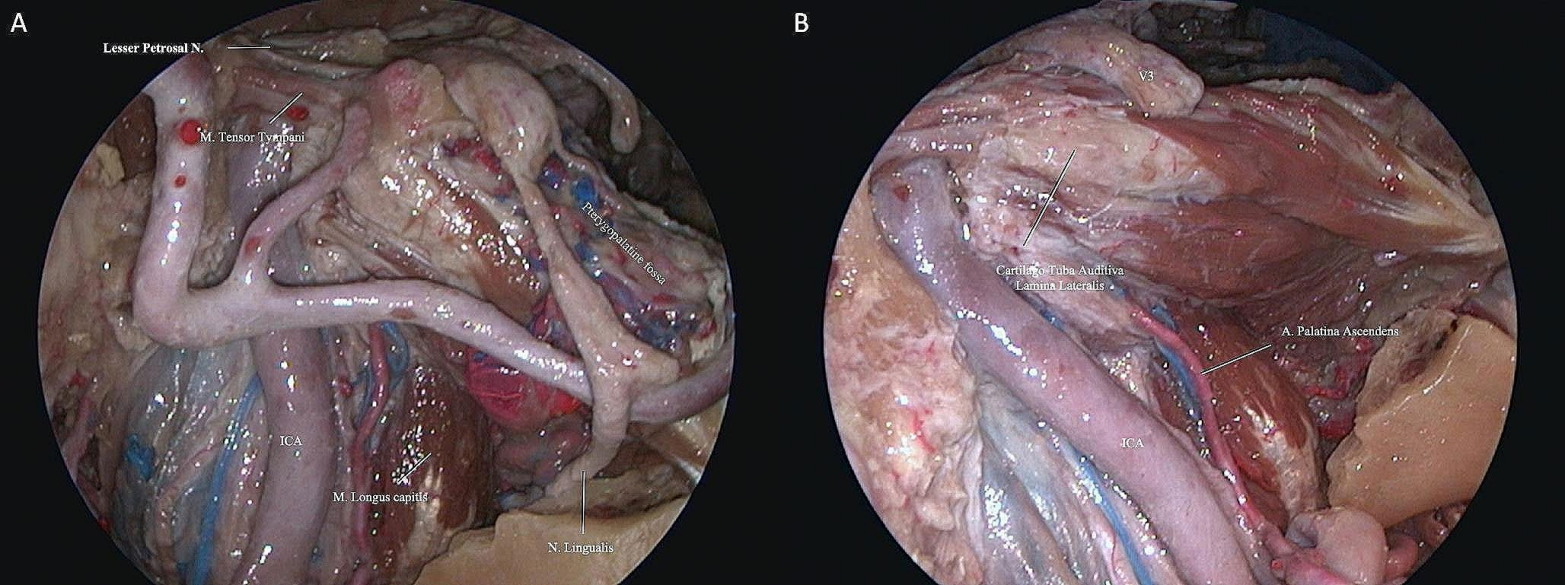
**(A)** The PT runs almost parallel to the pterygoid process from the PT sulcus to the nasopharynx through the scaphoid fossa and posteriorly, laterally, and superiorly from the nasopharynx to the middle ear and the horizontal segment of the internal carotid artery. Because there is less venous vascularity medial to the TVPM, this plane can be used to confirm the course of PT. **(B)** V3 and maxillary artery were resected to show the course of PT more clearly

**Fig. 7:**
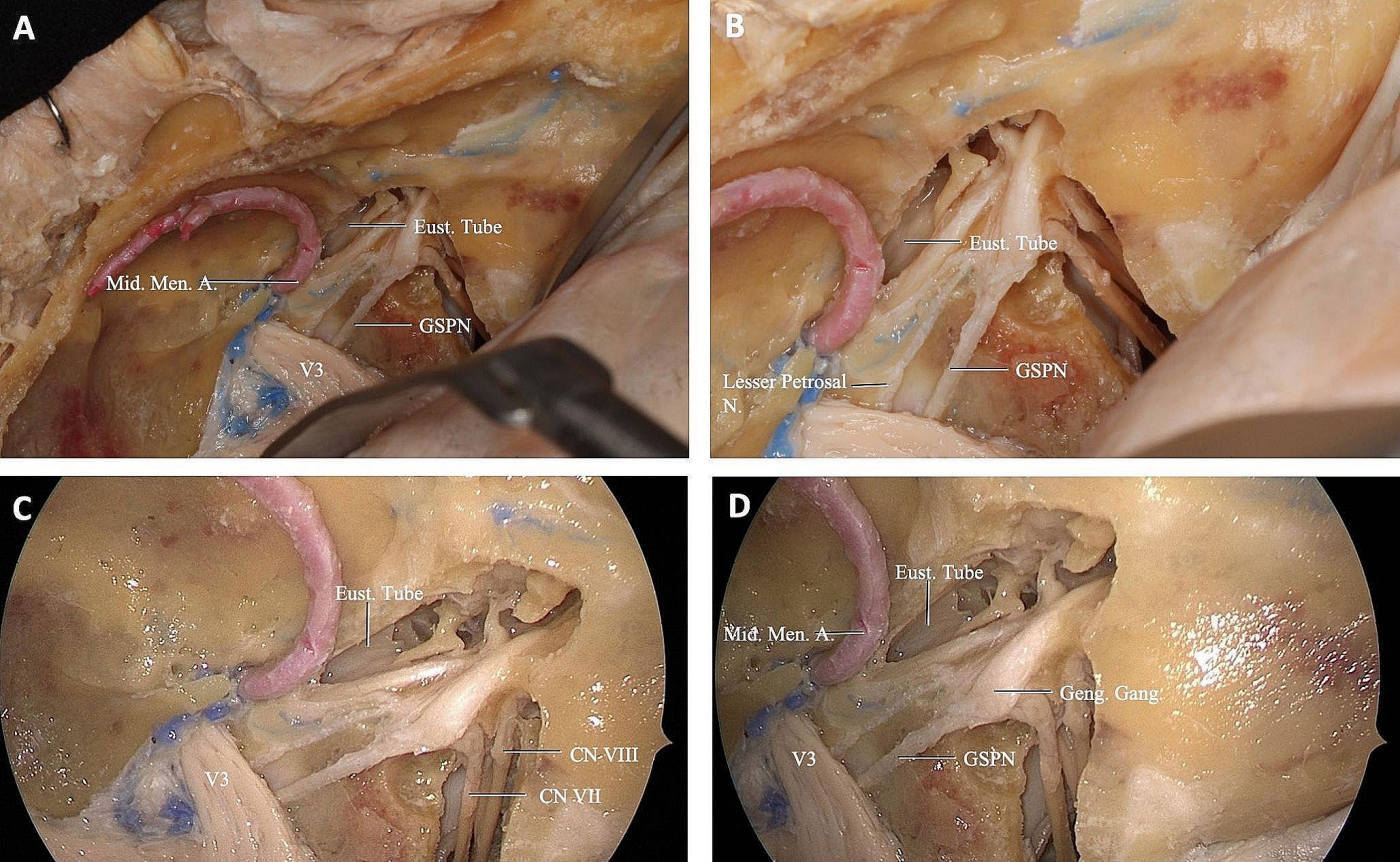
**(A)** Viewing the middle cranial fossa with a lateral (Subtemporal) approach; The petrous apex is shown in V3 and laterally. Osseous PT was demonstrated by extending the dissection laterally. **(B)** A zoomed view of the same dissection; TTM, GSPN, and PT were revealed in the middle cranial fossa. **(C)** Continuing the endoscope-guided dissection revealed osseous PT, V3 anterior to the TTM, and CN VII-VIII complex posteriorly within the internal acoustic canal. **(D)** Close-up oblique view of Fig. 7C. The relationship between PT and the CN VII tympanic segment was demonstrated

Next, the dissection was continued to expose the posterolateral, middle, and anteromedial parts of the PT. We found the posterolateral part was related to the greater wing of the sphenoid and PA, the middle part located above the FL, and the anteromedial part closely related to the pterygoid process. The mean of an obtuse angle between the midline and the left oblique line that is parallel to the PT trajectory and passes from pharyngeal PT orifice and tympanic orifice was 119.6 ± 3.81 and 119.8 ± 4.45 degrees on the left and right side, respectively (Table 1). PT ran from the PT sulcus to the nasopharynx through the scaphoid fossa and posteriorly, laterally, and superiorly from the nasopharynx to the middle ear and almost parallel to the horizontal segment of the internal carotid artery toward the pterygoid process. Removing the base of the pterygoid process exposed the pterygoid and scaphoid fossae, which contained the attachment of the MPM and TVPM. PT was not attached to these fossae and runs inferior to the scaphoid fossa. The venous plexus was found between the MPM and TVPM; however, less venous vascularity was found medial to the TVPM creating a plane that can be used to confirm the course of the PT (Fig. 6A, B). The TVPM was easily separated from the lateral surface of the PT. Lateralization of the TVPM clearly exposed the medial and lateral laminae of the cartilaginous tube and the LVPM. We found that the accessory meningeal artery passed superolateral to the TVPM and entered the skull base above the PT. We dissected the accessory meningeal artery to completely expose the attachment of the cartilaginous tube to the skull base. Bony dehiscence can often be observed on the medial wall of the osseous part of the PT that exposes the lateral wall of the ICA. The osseocartilaginous junction of the PT can be exposed from the superolateral direction, and the attachment of the LVPM was located just inferior to the junction. A fibrocartilaginous ring was then removed to facilitate the anterior translocation of the ICA, which allowed us to access and drill into the remaining PA. Infrapetrous approach to PA required the removal of the fibrocartilaginous tissue between the FL and PT to reach the anterior inferior PA. The ICA entered the cranial cavity through the carotid canal (CC) located in the petrous part of the temporal bone. The CC passed within the petrous bone, initially upward and then inward, and forward to the FL. Anterolateral to the carotid foramen there were two small canals, called the semicanal of the TTM and the semicanal of the PT, which passed under the tubal process of the tympanic part attached to the PA (Fig. 5A-C and Fig. 7). Anterior-medial to the semicanals, anterior and parallel to the horizontal segment of the CC, was the bony bed for the cartilaginous parts of the PT termed the PT sulcus. The anterior end of the PT sulcus was adjacent to the FL medially and scaphoid fossa laterally. At the FL, the PT and ICA were separated by fibrocartilaginous layers. Relevant radiologic measurements that were obtained on CT are shown in (Table 2).

**Table 2:** Radiological parameters of Eustachian tube and its associated anatomical structures in the infratemporal fossa

N: 50 (Female: 25 Male: 25)	Average ± SD (min, max) left side	Average ± SD (min, max) right side
The distance from VC to the same side base of the medial pterygoid plate base (coronal plane) (mm)	8.1 ± 3.25 (1.9, 14.5)	8.4 ± 3.36 (3.3, 14.7)
The direct distance from the ventral opening of the VC to the same side FR (coronal plane) (mm)	7.3 ± 2.97 (3.2, 13.4)	6.98 ± 3.0 (1.6, 13.5)
The distance from the ventral VC opening to the optic canal (anterior skull base) (coronal plane) (mm)	18.8 ± 1.9 (14.5, 24.2)	19.1 ± 2.02 (14.0, 23.2)
The distance from the nasal vestibule to the PT orifice (axial plane) (mm)	73.2 ± 4.21 (62.9, 81.4)	72.7 ± 4.39 (64.8, 84.6)
The length of VC from anterior to posterior (axial plane) (mm)	11.9 ± 2.07 (7.1, 15.7)	12.3 ± 1.91 (8.5, 16.8)
The length of the PT (from the nearest point to the pharynx of the pharyngeal PT orifice to the tympanic orifice) (axial plane) (mm)	45.07 ± 2.45 (39.5, 51.5)	44.7 ± 2.32 (39.6, 50.2)
An obtuse angle between the midline and the oblique line which is parallel to the PT trajectory and passes from pharyngeal PT orifice and tympanic orifice (axial plane) (degree)	122.7 ± 4.53 (112.2, 131.3)	124.1 ± 4.4 (113.4, 132.8)

## Discussion

Several endoscopic approaches and variable surgical corridors have been described to expose the PT [14, 16, 20, 23]. Selecting a suitable approach depends on the location of the lesion, relationship with important neurovascular structures, and careful pre-operative review of radiological data [5, 7, 17]. In this combined cadaveric and radiologic study, we showed the relevant anatomic considerations for ETETA. There is currently limited literature on such endoscopic approaches, including limited anatomical data on the approach to the ITF and PA [6, 16]. The nasal cavity, sphenoidal sinus, PT, and surrounding structures can provide surgical corridor to the MCF, ACF [6, 14, 17]. Endoscopic approaches can be more effective to accessing the brainstem, ventral skull base, ACF, and MCF than the lateral transcranial approaches, as retraction of the brain and neurovascular structures can be decreased [1, 10, 14, 17]. However, precise identification of the important anatomical landmarks is required to avoid crucial neurovascular structures and safely perform surgery [3, 5, 14, 17]. We demonstrate these pertinent landmarks in planning ETETA for access to ITF and PA.

The PT was first described by the Italian anatomist Bartolomeo Eustachi [9, 13]. The PT has traditionally been divided into a medial osseous and a lateral cartilaginous portion and is located above the foramen lacerum. Understanding the complex anatomy of PT is possible by defining the corridor to the ITF and PA in reference to precise anatomical landmarks [12, 14, 22]. The ETETA presents several surgical challenges and radiologic analysis can be helpful prior to surgery. On axial CT, the mean distance from the nasal vestibule to the PT orifice was 73.2 ± 4.21 mm on the left and 72.7 ± 4.39 mm on the right side. The mean length of the left PT was 45.07 ± 2.45 mm and the mean length of the right PT was 44.7 ± 2.32 mm. Similar results were obtained in cadaveric dissection with a mean length of the PT of 41.7 ± 2.3 mm and 42.7 ± 0.32 mm and the mean shortest distance from the nasal vestibule to the PT orifice of 76 ± 4.58 mm and 75 ± 3.6 mm on the left and right side, respectively. An obtuse angle between the midline and the oblique line, which is parallel to the PT trajectory and passes from pharyngeal PT orifice and tympanic orifice gives an idea of the PT trajectory and had a mean of approximately 120 degrees, similar between dissection and CT.

VC is also a crucial landmark in this surgery [12]. On coronal CT, the mean distance from VC to the same side base of the MPP base on coronal CT was 8.1 ± 3.25 mm and 8.4 ± 3.36 mm, mean direct distance from the ventral opening of the VC to the same side FR was 7.3 ± 2.97 mm 6.98 ± 3.0 mm, and mean distance from the left ventral VC opening to the optic canal (anterior skull base) was 18.8 ± 1.9 mm and 19.1 ± 2.02 mm on the left and right sides, respectively. The mean length of VC from anterior to posterior was 11.9 ± 2.07 mm on the left and 12.3 ± 1.91 mm on the right side on axial CT. Our endoscopic dissection from an inferior approach showed that the long axis of the PT on the axial plane corresponds to the line passing through the medial opening of the osseous part of the PT and the dorsal opening of the VC. The mean (SD) direct distance from the ventral opening of the VC to the PT orifice was 22.9 ± 0.51 mm and 22.7 ± 0.65 mm on the left and right side, respectively. The VC was directed posteriorly toward the second genu of the ICA. Once the VC was exposed, the base of the pterygopalatine plates (PTPs) was identified immediately laterally. The VN was transected just proximal to its junction with the sphenopalatine ganglion to expose the lateral pterygoid plate (LPP). On examination, the mean shortest distance from the ventral VC opening to the optic canal was 22.4 ± 0.9 mm and 21.4 ± 0.55 mm on the left and right side, respectively.

Finally, the lateral limit of the ETETA can be accepted as the jugular tubercle, jugular fossa, internal auditory canal, and posterior vertical segment of the ICA. The lateral limit of these approaches is still controversial and needs more investigation [14, 18, 19]. ETETA to the ACF and MCF are very challenging, and the VN, VC, and the close relationship with other foramina and neurovascular structures are not well known. Our results provide guidance to successfully navigating this challenging approach.

## Conclusions

We have shown that endoscopic exposure of the ITF and PA via the PT is feasible and safe. Transecting the PT and/or resection to maximize access has been associated with various complications. Safe zones for PT mobilization were identified for providing comparable access with suitable degrees of exposure. Safely performing this procedure requires an in-depth understanding of the relationship between the PT and its surrounding structures. Our cadaveric dissection and radiologic analysis show the critical steps to successfully navigating this challenging approach.

## Data Availability

No datasets were generated or analysed during the current study.

## Abbreviations

ACF: Anterior cranial fossa
CC: Carotid canal
CT: Computerized tomography
ETA: Endoscopic transnasal approach
ETETA: Endoscopic trans-eustachian tube approach
FL: Foramen lacerum
FO: Foramen ovale
FR: Foramen rotundum
ICA: Internal carotid artery
IJV: Internal jugular vein
IMA: Internal maxillary artery
ITF: Infratemporal fossa
LPM: Lateral pterygoid muscle
LPP: Lateral pterygoid plate
LVPM: Levator veli palatini muscles
MCF: Middle cranial fossa
MPM: Medial pterygoid muscle
MPP: Medial pterygoid plate
PA: Petrous apex
PphICA: Parapharyngeal internal carotid artery
PPF: Pterygopalatine fossa
PT: Pharyngotympanic tube
SD: Standard deviation
TT: Torus tubarius
TTM: Tensor tympani muscle
TVPM: Tensor veli palatini muscles
VC: Vidian canal
VN: Vidian nerve
V2: Maxillary nerve
V3: Mandibular nerve
3D: Three-dimensional
